# Central Venous Catheter Misplacement Presenting With Parotid Gland Swelling: A Case Report

**DOI:** 10.7759/cureus.66245

**Published:** 2024-08-05

**Authors:** David Mestre, Francisco Gil

**Affiliations:** 1 Intensive Care Unit, Hospital José Joaquim Fernandes, Beja, PRT

**Keywords:** parotid gland swelling, metformin-associated lactic acidosis, external jugular vein thrombosis, central venous catheter (cvc), catheter-related thrombosis, catheter-related complications

## Abstract

Central venous catheter (CVC) placement is a routine procedure in ICUs but can be associated with various complications, including misplacement and thrombosis. We present a rare case of parotid gland enlargement due to catheter-related thrombosis of the external jugular vein following ultrasound-guided placement through the subclavian vein in an 84-year-old woman. This case was managed with systemic anticoagulation and catheter removal. It emphasizes the importance of confirming correct CVC tip positioning and highlights the need for a post-procedure chest X-ray.

## Introduction

Central venous catheter (CVC) placement is a common procedure in ICUs. It is frequently indicated for vasopressor or inotropic infusion, renal replacement therapy, and hemodynamic monitoring. CVC placement can be associated with early and late complications. Early complications include vascular injury, hematoma, bleeding, pneumothorax, air embolus, arrhythmia, and misplacement, while complications such as infection and thrombosis can occur after a few days [1].

There are two fundamental techniques for CVC placement: insertion can be guided by anatomical landmarks or ultrasound. Recently, with the broader availability of ultrasonography and aiming to decrease risks, there has been an increase in ultrasound-guided procedures. With the widespread use of ultrasound-guided procedures, some authors argue for the removal of the routine post-CVC placement chest X-ray [2,3]. However, neither technique is free from complications.

Infectious conditions are the most frequent causes of parotid gland swelling. Non-infectious causes are rare and include a variety of inflammatory conditions, such as autoimmunity. Uncommon causes include trauma, surgery, radiation therapy, and venous vascular abnormalities [4].

We present a rare case of parotid gland enlargement due to catheter-related thrombosis of the external jugular vein following ultrasound-guided placement.

## Case presentation

An 84-year-old woman with well-controlled diabetes mellitus, managed with metformin, was brought to the ED due to a behavioral change that began 12 weeks ago, increasing in severity.

In the ED, the patient reported persecutory thoughts without any other alterations on the medical exam. Laboratory analysis revealed normal liver and kidney function and ions (sodium, potassium, and chloride). Infection was excluded due to negative C-reactive protein (CRP) and procalcitonin (PCT) values. Cerebral imaging studies, including CT and MRI, showed no acute findings. The patient was admitted to the Psychiatry Department (PD) for further investigation. After a psychiatric evaluation, she was started on olanzapine and promethazine, while continuing metformin 1000 mg twice a day.

On day seven, the patient developed tachypnea and anuria. Arterial blood gas on room air revealed high anion gap metabolic acidemia (pH 6.89, pCO2 17 mmHg, pO2 116 mmHg, bicarbonate 6 mmol/L, lactate 12 mmol/L, potassium 8.3 mmol/L, sodium 129 mmol/L, chloride 95 mmol/L, anion gap 28 mEq/L, glucose 132 mg/dL). 

Subsequently, sinus bradycardia was diagnosed. The medical staff started emergent treatment for hyperkalemia with an insulin-glucose infusion and heart-protecting measures with calcium chloride. Blood analysis showed elevated serum urea (255 mg/dL, reference range 17-43mg/dL) and serum creatinine (11.40 mg/dL, reference range 0.7-1.3), more than three times the baseline. Serum metformin levels were toxic (24 mg/L, toxicity risk above five mg/L). Inflammatory biomarkers (CRP 0.3 mg/dL, reference range <0.5 mg/dL, and PCT 0.01 ng/mL, reference range <0.05 ng/mL) remained negative. Blood and urine cultures were also negative (Table 1). CT excluded urinary obstruction. The diagnosis of metformin-associated lactic acidosis was made, and the patient was transferred to the ICU.

**Table 1:** Laboratory findings. Laboratory findings showed an acute kidney injury, a toxic level of metformin, and no evidence of infection.

	Results	Normal range
Serum urea (mg/dL)	255	17-43
Serum creatinine (mg/dL)	11.40	0.7-1.3
Serum metformin (mg/L)	24	Toxicity risk >5
C-reactive protein (mg/dL)	0.3	<0.5
Procalcitonin (ng/mL)	0.01	<0.05
Blood cultures	Negative	Negative
Urine culture	Negative	Negative

**Figure 1:**
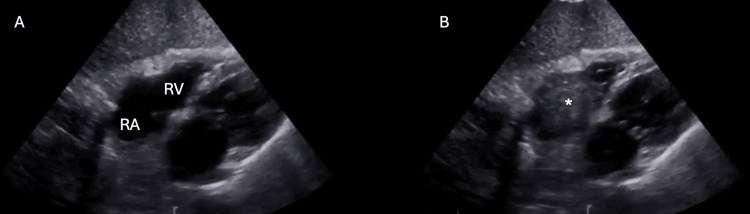
Subcostal echocardiography view with bubble test. A: Subcostal echocardiography view before the bubble test showing the Right Atrium (RA) and Right Ventricle (RV). B: Subcostal echocardiography view during the bubble test showing flow through the right heart (*).

In the ICU, a CVC was inserted in the right subclavian vein for drug infusion and another in the right femoral vein for continuous renal replacement therapy (CRRT), both using an ultrasound-guided technique. After CVC placement, an echocardiogram with a bubble test was performed to confirm the CVC's position in the venous system (Figure 1).

The patient started vasopressors (norepinephrine maximum dose of 0.22mcg/Kg/min) and Continuous Venovenous Hemodiafiltration with regional citrate anticoagulation (effluent dose of 30mL/Kg/h). After 48 hours, the patient showed recovery of intrinsic renal function without metabolic abnormalities, allowing discontinuation of CRRT and vasopressors.

**Figure 2:**
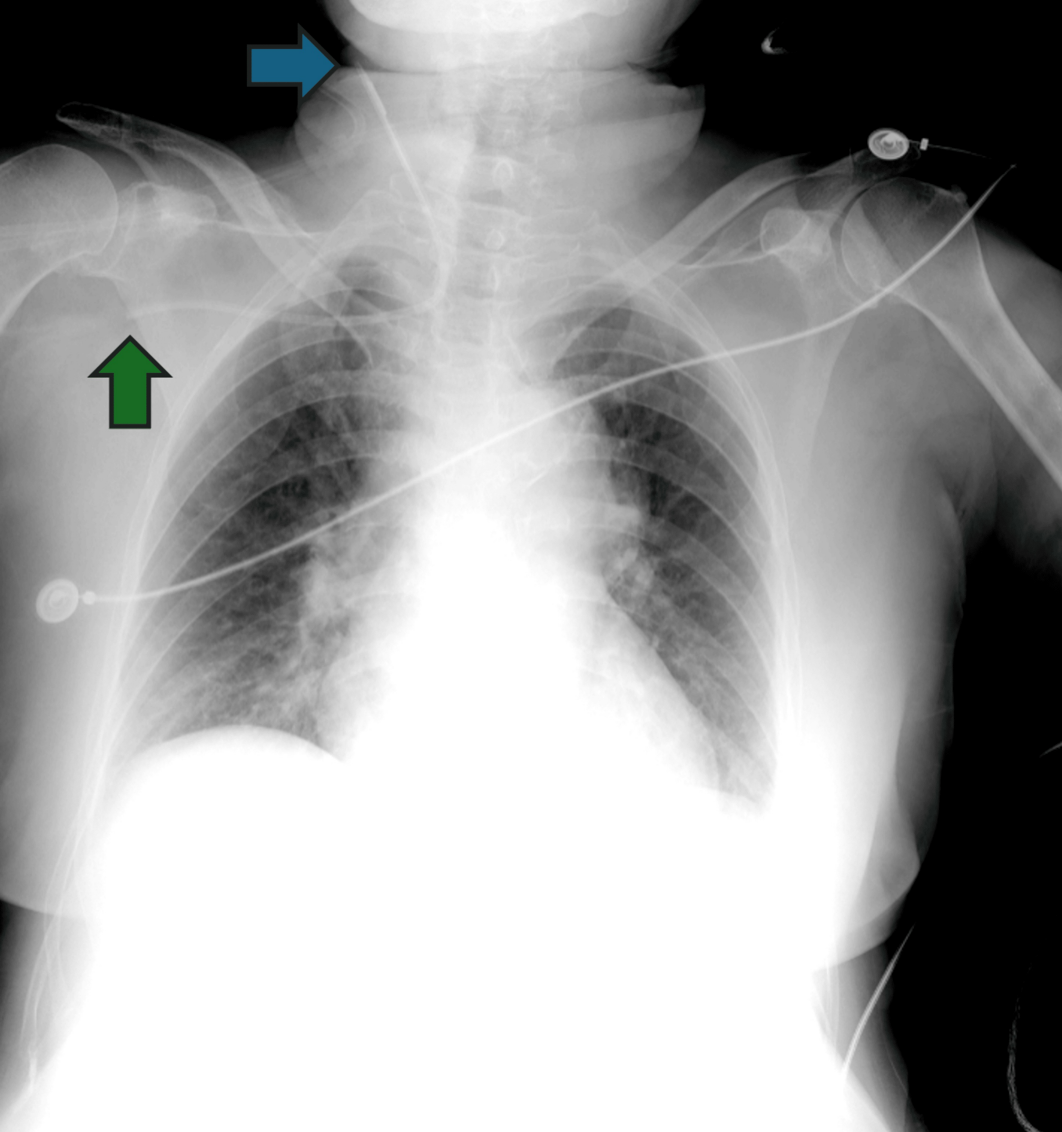
Chest X-ray. The chest X-ray revealed a central venous catheter with the entrance in the right subclavian vein (green arrow) and a misplaced tip (blue arrow).

**Figure 3:**
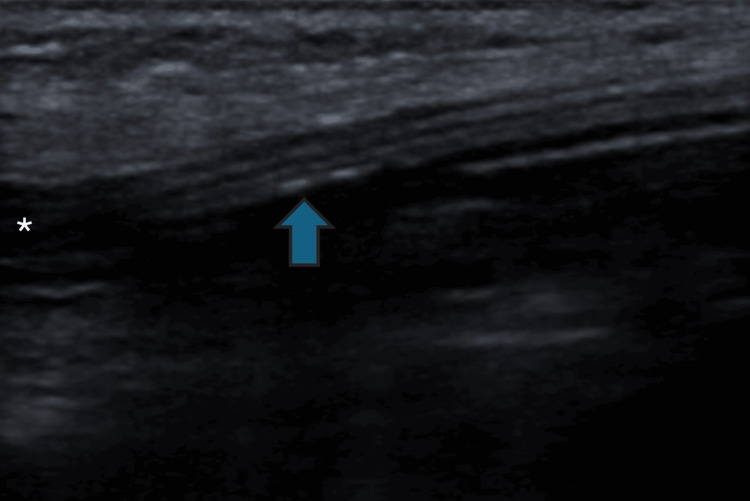
Ultrasound of right external jugular vein. The ultrasound shows the tip of the central venous catheter (blue arrow) inside the right external jugular vein (*).

On day nine, the patient complained of pain at the right jaw angle with associated swelling. A chest X-ray revealed a misplaced CVC tip (Figure 2). An ultrasound showed the catheter tip in the right external jugular vein (Figure 3) with indirect signs of thrombosis by a non-collapsible vein, and swelling of the parotid gland.

Catheter-related thrombosis of the right external jugular vein was diagnosed, and therapeutic systemic anticoagulation with enoxaparin was initiated. The CVC was removed, and the catheter tip and blood were sent for culture; all were negative. No increase in inflammatory biomarkers was observed. After four days, the swelling resolved, and the patient was discharged from the ICU to the PD. On day 17, the patient was discharged from the hospital on oral anticoagulants, with no recurrence of parotid gland swelling and low inflammatory biomarkers.

## Discussion

Venous thrombosis is a well-known complication of venous cannulation. The rates of asymptomatic deep venous thrombosis are around 19% in studies using Doppler ultrasound, but they increase to 41% when venography is used to screen patients [5].

The risk factors for catheter-related thrombosis include those related to the patient and those dependent on the technique. Jugular CVCs are associated with a greater risk of thrombosis compared to subclavian CVCs. Multi-lumen CVCs, larger CVCs, and misplaced CVCs also increase the risk [6]. In the case we present, besides the CVC being placed through the subclavian vein, the tip misplacement into the external jugular vein brought an additional thrombotic risk.

Despite some authors claiming that the safety of ultrasound-guided cannulation can eliminate the need for routine chest X-rays after CVC placement [2,3], the chest X-ray has the advantage of easily detecting tip position. In contrast, ultrasound-guided cannulation of the superior vena cava territory generally only allows indirect tip detection unless the wire or catheter is advanced deep into the superior vena cava and right atrium. The echocardiogram we performed showed no indirect signs of catheter misplacement or other complications.

The retromandibular vein drains the parotid gland. It splits into two branches: the posterior branch joins the posterior auricular vein, forming the external jugular vein, while the anterior branch joins the facial vein, a tributary of the internal jugular vein [7,8].

Internal jugular vein thrombosis is a well-known consequence of infectious parotitis, also known as Lemierre syndrome [8]. However, venous thrombosis leading to parotid gland enlargement is extremely rare. There have been a few reported cases of retromandibular vein venous injuries causing parotid gland enlargement [4]. In the case we present, infection was ruled out as both inflammatory biomarkers (CRP and PCT) and microbiologic cultures were negative. After removing the CVC and starting enoxaparin, the parotid swelling resolved without the need for antibiotics or immunosuppressive therapy.

Despite all the evidence available and concerns about radiation exposure and costs, this case shows the importance of performing a chest X-ray after insertion of a CVC, even when an ultrasound-guided technique was used.

A routine post-procedure chest X-ray could help prevent the reported iatrogenic complication. Vascular ultrasound of the neck's great vessels could also identify CVC misplacement in this patient but might not detect tip misplacement in inaccessible veins. Therefore, a chest X-ray might be helpful after CVC insertion.

## Conclusions

We report a rare case of catheter-related thrombosis of the external jugular vein due to CVC misplacement presenting as parotid gland swelling. Although CVC placement under ultrasound guidance is safer, it does not guarantee correct positioning. Clinicians should maintain a high index of suspicion and utilize appropriate imaging techniques for timely identification and management of such complications. Thus, a chest X-ray still plays a crucial role.
